# Transcriptome analysis of genes involved in secondary cell wall biosynthesis in developing internodes of *Miscanthus lutarioriparius*

**DOI:** 10.1038/s41598-017-08690-8

**Published:** 2017-08-22

**Authors:** Ruibo Hu, Yan Xu, Changjiang Yu, Kang He, Qi Tang, Chunlin Jia, Guo He, Xiaoyu Wang, Yingzhen Kong, Gongke Zhou

**Affiliations:** 1 Key Laboratory of Biofuels, Qingdao Engineering Research Center of Biomass Resources and Environment, Qingdao Institute of Bioenergy and Bioprocess Technology, Chinese Academy of Sciences, Qingdao, 266101 P. R. China; 2 Shandong Institute of Agricultural Sustainable Development, Jinan, 250100 P. R. China; 3 Key laboratory of Tobacco Genetic Improvement and Biotechnology, Tobacco Research Institute of Chinese Academy of Agricultural Sciences, Qingdao, 266101 P. R. China

## Abstract

*Miscanthus* is a promising lignocellulosic bioenergy crop for bioethanol production. To identify candidate genes and regulation networks involved in secondary cell wall (SCW) development in *Miscanthus*, we performed *de novo* transcriptome analysis of a developing internode. According to the histological and *in-situ* histochemical analysis, an elongating internode of *M. lutarioriparius* can be divided into three distinct segments, the upper internode (UI), middle internode (MI) and basal internode (BI), each representing a different stage of SCW development. The transcriptome analysis generated approximately 300 million clean reads, which were *de novo* assembled into 79,705 unigenes. Nearly 65% of unigenes was annotated in seven public databases. Comparative profiling among the UI, MI and BI revealed four distinct clusters. Moreover, detailed expression profiling was analyzed for gene families and transcription factors (TFs) involved in SCW biosynthesis, assembly and modification. Based on the co-expression patterns, putative regulatory networks between TFs and SCW-associated genes were constructed. The work provided the first transcriptome analysis of SCW development in *M. lutarioriparius*. The results obtained provide novel insights into the biosynthesis and regulation of SCW in *Miscanthus*. In addition, the genes identified represent good candidates for further functional studies to unravel their roles in SCW biosynthesis and modification.

## Introduction

*Miscanthus* is a perennial C4 rhizomatous grass originated from eastern and southern Asia. The *Miscanthus* genus contains more than 14 species, of which four major species (i. e., *Miscanthus sinensis*, *Miscanthus floridulus*, *Miscanthus sacchariflorus*, and *Miscanthus lutarioriparius*) are most extensively distributed in China^1^. Recently, *Miscanthus* has been considered one of the most promising bioenergy crops for lignocellulosic bioethanol production owing to its superior characteristics such as high biomass yield, wide suitability to cultivation on marginal land, and low fertilizer requirement^2–4^. As a widely distributed species in central China, *M. lutarioriparius* has been paid particular attention because of its overwhelmingly higher biomass yield, vigorous growth habitus and wide adaptation to various abiotic stresses^5, 6^.

One of the major steps in bioethanol production from lignocellulosic biomass is the release of fermentable sugars embedded in secondary cell walls (SCWs)^7^. SCW is mainly composed of cellulose, hemicellulose and phenolic polymer lignin, which interact and cross-link with each other to form a rigid and complex network^8, 9^. As biomass consists predominately of SCWs, thus the compositions and cross-linking among different components of SCW are of significant impact on biomass recalcitrance to saccharification^7^. In this perspective, a thorough understanding of key genes involved in SCW biosynthesis and modification lays the prerequisite for tailor-designing of optimized biomass characteristics in bioenergy crop breeding.

Reverse genetics analysis and biochemical assays have revealed a subset of genes involved in SCW biosynthesis and modification, especially in dicot model species *Arabidopsis* ^10^. Cellulose is comprised of linear β-1,4-glucan chains. It is synthesized at the plasma membrane by members of cellulose synthase (CESA) enzymes. The deposition of cellulose in SCWs requires the cooperative functions of CESA4, CESA7 and CESA8^11^. Hemicellulose usually consists of linear β-1,4-linked backbones, which are usually substituted with various side chains. Based on side chain substitutions, hemicellulose can be classified into several categories including heteromannans, xyloglucans, heteroxylans, and β-(1,3;1,4)-mix-linked glucans^12^. Xyloglucans are the predominant hemicelluloses in the primary cell walls of dicots, while xylans are the most abundant hemicellulose components in SCWs of dicots and both primary and secondary walls of commelinid monocots^12^. A number of genes from the CESA-like (CSL) family have been revealed to participate in the biosynthesis of various hemicellulosic polysaccharides. For instance, several CSLA family genes from glycosyltransferase (GT) family 2 have been demonstrated to be responsible for the biosynthesis of the glucomannan backbone^13^. Several members of the CSLC family are implicated in the biosynthesis of the xyloglucan backbone^14^. Members from CSLF and CSLH families have been revealed to participate in the biosynthesis of mixed-linkage β-glucan^15, 16^. In addition, a number of GTs from other families have also been identified to be involved in xylan biosynthesis. For example, IREGULAR XYLEM (IRX) 9 and IRX14 from GT43 family, and IRX10 from GT47 family are required for the elongation of the xylan backbone^17–20^, whereas FRAGILE FIBER (FRA) 8/IRX7 from GT47 family, IRX8 and PARVUS from GT8 family are involved in the synthesis of the reducing-end tetrasaccharide of xylan^21–23^. Lignin is a complex aromatic polymer synthesized from phenylpropanoid precursors. It primarily consists of three monomeric subunits, i.e., p-hydroxyphenyl (H), guaiacyl (G), and syringyl (S)^24^. Lignin biosynthesis has been well-characterized and almost all the genes for each step of the monolignol biosynthetic pathway have been identified by utilizing reverse genetic approaches and biochemical assays in different plant species^24^. Besides these major components, SCWs also contain a minor proportion of structural proteins. These cell wall proteins are trapped in the complex networks formed by cell wall polysaccharides and lignin. Although cell wall proteins are minor components in SCWs, they play essential roles in maintaining cell wall strength and confer appropriate properties to cell walls^25^. Among the cell wall proteins, expansions are considered one of the major regulators of cell wall loosening and extensibility during plant cell growth^26^. In addition, extensins and arabinogalactan-proteins (AGPs), which belong to the hydroxyproline-rich glycoprotein (HRGP) family, also play essential roles in the cell wall assembly and modification^27^.

Biochemical analysis has also led to the identification of various types of enzymes associated with cell wall polymer degradation and remodeling. Among the numerous enzymes, one of the most characterized is the glycosyl hydrolases (GHs). GHs constitute large gene families in plants and have been implicated to be involved in the remodeling and rearrangement of plant cell walls^28^. GH9 family represents one of the best characterized plant GH families. The KORRIGAN (KOR) protein from GH9 family has been revealed to play an important role in cellulose biosynthesis. It might function in the cleavage of a sterol-cellodextrin precursor or assist in the assembly of glucan chains in the growing microfibrils^29, 30^. In addition, several members from GH10 and GH11 families are considered to catalyze the modification and degradation of hemicelluloses (e.g., xyloglucan and xylan)^31, 32^.

Meanwhile, considerable progress has been gained toward the understating of the transcriptional regulation of SCW biosynthesis in the last decade. The NAC (NAM, ATAF1/2, and CUC2) transcription factors (TFs) function as master switches in the regulation of SCW biosynthesis^33^. Several NAC TFs, such as SECONDARY WALL ASSOCIATED NAC DOMAIN PROTEIN 1 (SND1)/NAC SECONDARY WALL THICKENING PROMOTING FACTOR 3 (NST3), have been shown to orchestrate the expression of a cascade of down-stream TFs (e.g., MYB46 and MYB83), which cooperatively regulate the expression of SCW biosynthesis genes^34^.

Although significant progress in SCW biosynthesis has been made in the model species *Arabidopsis* and rice, much less is known about the molecular mechanisms underlying SCW biosynthesis, assembly and modification in bioenergy crop *Miscanthus*. *M. lutarioriparius* is a diploid (2n = 2x = 38) with a complex genome estimated to be approximately 2000 Mb^35, 36^. The genome sequence of *M. lutarioriparius* has not been released yet, which imposes great difficulties to genome-wide studies on SCW-related genes in this species. However, with the advancement of the next-generation sequencing technology and ever-decreasing of sequencing cost, *de novo* transcriptome analysis without a reference genome provides an alternative means to the identification of genes involved in many important physiological processes. Transcriptome profiling has been successfully utilized to identify genes involved in SCW biosynthesis in diverse plant species, including poplar^37^, maize^38, 39^, sorghum^40^, *Medicago* ^41^ and switchgrass^42^. However, despite the importance of optimized SCW properties to the improvement of saccharification efficiency in bioethanol production, such transcriptome analysis aimed to explore genes involved in SCW development is still lacking in *Miscanthus*.

In this study, we carried out transcriptome analysis of *M. lutarioriparius* using three consecutive internode segments, representing different stages of SCW development, with an attempt to advance the understanding of SCW biosynthesis and regulation in *Miscanthus*. This work described a global transcriptome profiling of genes during SCW biosynthesis and identified candidate genes and regulation networks underlying SCW metabolism. To the best of our knowledge, this report represents the first transcriptome analysis of SCW biosynthesis in *Miscanthus*. The results obtained deepen our understanding of SCW biosynthesis in *Miscanthus*. In addition, the genes identified provide good candidates for future functional studies to improve the biomass properties of *Miscanthus*.

## Results

### Cell wall development in three consecutive segments of elongating internodes

**Figure 1:**
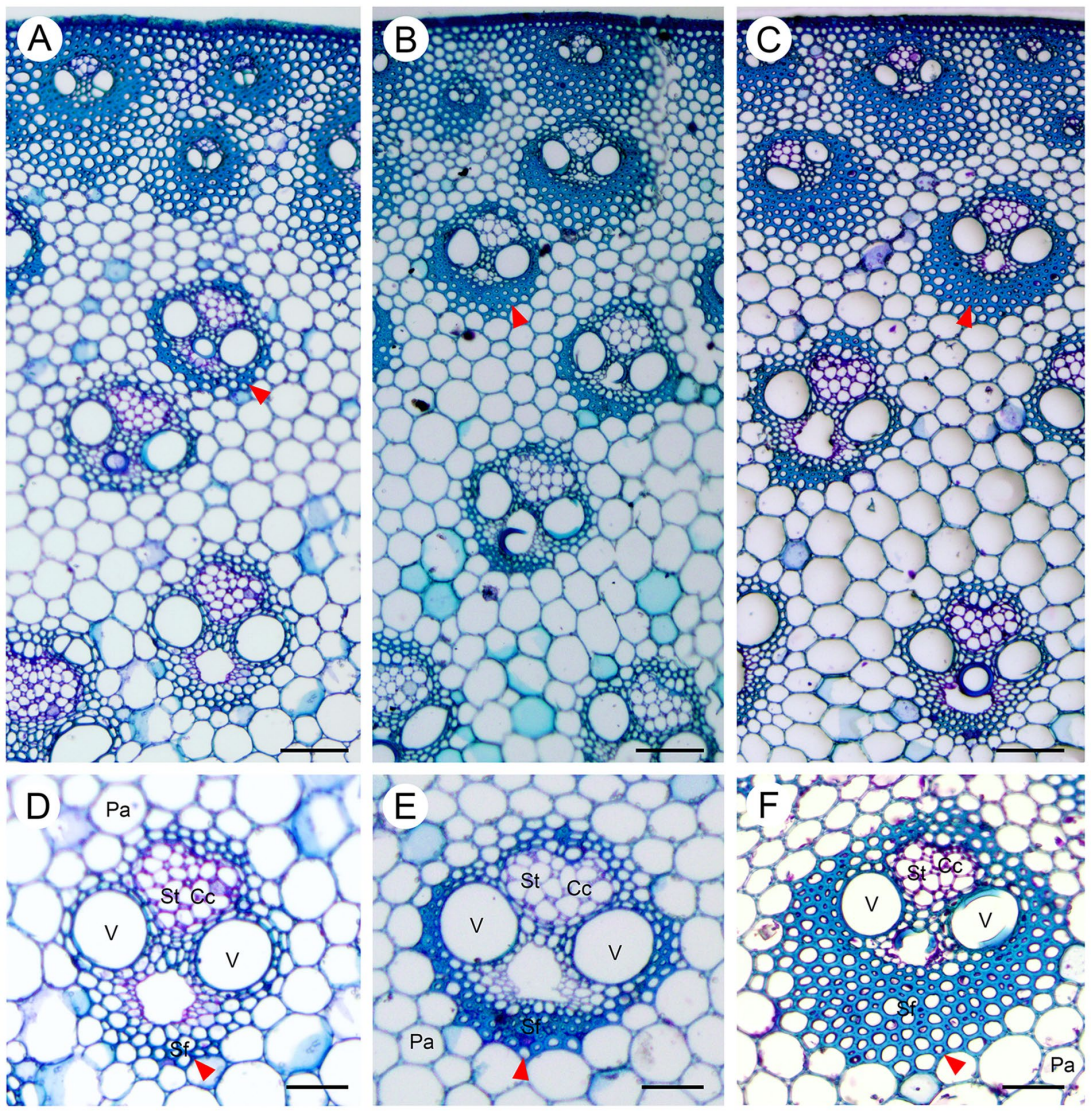
Anatomy of an elongating internode of *M. lutariorioparius*. Sections from three segments of the 2^nd^ internode from top were stained with Toluidine Blue O and observed under bright field. (**A**) upper internode (UI), (**B**) middle internode (MI), (**C**) basal internode (BI), (**D**,**E** and **F**) represent the magnified view of a vascular bundle in A, B, and C, respectively. Arrow head indicates the thickened fiber cells. Cc, companion cells, Pa, parenchyma, Sf, sclerenchyma fiber, St, sieve tube, V, vessel. Scale bar 100 μm in (**A–C**), and 50 μm in (**D**–**F**).

To understand the dynamic changes of SCW development in *M. lutarioriparius*, we firstly analyzed the cell wall morphology of three consecutive segments of an elongating internode (2^nd^ internode from top). The stem sections were stained with Toluidine Blue O to visualize the different cell types using brightfield microscopy (Fig. 1). The upper internode (UI) contained immature vascular bundles with protoxylem and un-thickened fibers and vessels. Cell wall thickening was only observed in cells proximate to the cortex. The middle internode (MI) contained fully developed vascular bundles with modestly thickened xylem fibers and vessels. The vascular bundles have significantly expanded in width. The basal internode (BI) also had well-developed secondary phloem tissues and secondary xylems, but the cell wall thickening was more pronounced than that of the MI.

**Figure 2:**
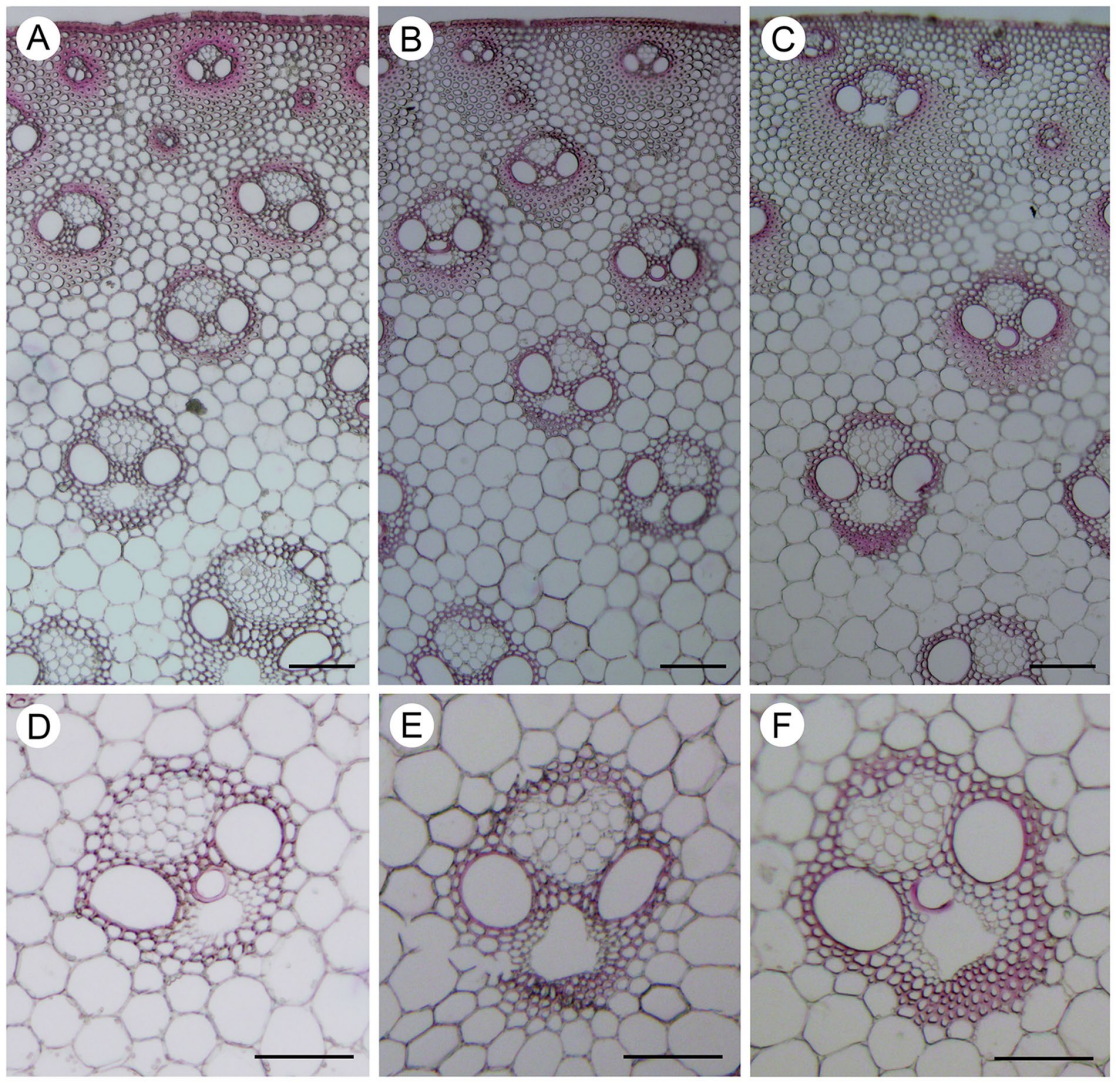
Lignification in an elongating internode of *M. lutariorioparius*. Lignification in three segments of an elongating internode (2^nd^ from top) was shown by Phloroglucinol-HCl staining (red color). (**A**) upper internode (UI), (**B**) middle internode (MI), (**C**) basal internode (BI), (**D**,**E** and **F**) are enlarged view of a specified vascular bundle in (**A**,**B** and **C**) respectively. Bar = 100 μm.

Phloroglucinol staining was carried out to visualize lignin in three different internode sections (Fig. 2). Only low levels of lignin were present in vascular bundles adjacent to cortex in the UI, while a modest number of cell walls have undergone lignification in the MI. In contrast, the BI cell walls accumulated significant amounts of lignin compared with those in the UI and MI.

**Figure 3:**
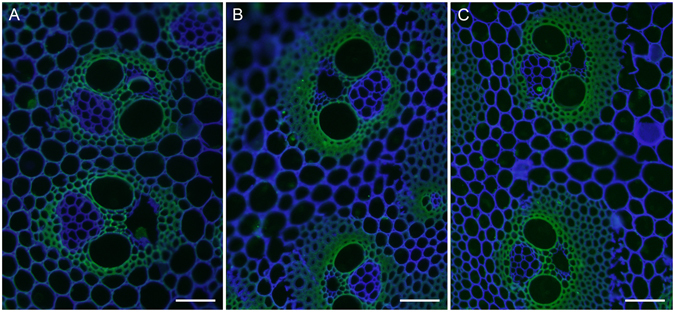
Immunolabeling of crystalline cellulose in an elongating *M. lutarioriparius* internode. The crystalline cellulose epitope in three sections of *M. lutarioriparius* internode was immunolabeled with CBM3a. The sections were counterstained with Calcofluor. (**A**) upper internode (UI), (**B**) middle internode (MI), (**C**) basal internode (BI). Bar = 100 μm.

We further examined the presence of cellulose in different internode sections with the carbohydrate-binding module CBM3a (Fig. 3), which specifically recognizes crystalline cellulose^43^. The fluorescence signal was detected in the SCWs of vascular bundles in the UI. In contrast, the more developed SCWs in the vascular bundles of the MI exhibited much stronger fluorescence. More intense fluorescence signal was detected in the SCWs of vascular bundles in the BI.

The distribution xylan epitopes in different internode sections was further determined using the LM10 monoclonal antibody (Supplemental Fig. 1), which recognizes xylan backbone with low- or non-substitutions^44^. Weak labelling signal was present in a few layers of thickened cells of the vascular bundles in the UI, while strong fluorescence signal was observed in the SCWs of vascular bundles in the MI. Even more intense fluorescence signal was detected in remarkably thickened SCWs in the BI.

Judged from these results, it can be concluded that the three internode segments contain various cell types with different extents of SCW thickening. The UI contains few SCWs, whereas the MI is rapidly synthesizing considerable amounts of SCWs, and the BI has almost completed SCW thickening. Thus the internode segments can be possibly utilized as a model system to study the SCW development in *M. lutarioriparius*.

### Transcriptome assembly and functional annotation

To identify genes involved in SCW development in *M. lutarioriparius*, the transcriptome profiling of three internode segments was performed using Illumina RNA-Seq. Approximately 12 gigabase (GB) clean data was obtained for each segment. Totally, the clean reads were assembled into 143,456 contigs with a mean length of 1,083 bp. These contigs were further assembled into a total of 79,705 unigenes with a mean length of 765 bp.

The assembled unigenes were annotated using BLASTX searches against seven publicly available databases with an E-value threshold of 1E-5. In total, 51,186 unigenes (64.2%) have significant BLAST hits in at least one of the databases, whereas 28,519 genes (35.8%) remain non-annotated. Among the annotated unigenes, 60.2% of unigenes had E-values higher than 1E-45 with corresponding sequences in the databases, whereas 39.8% of unigenes showed E-values ranging from 1E-45 to IE-5 (Supplemental Fig. 2A). About 64.8% of unigenes exhibited similarities higher than 80%, while 9.6% matched hits had similarities ranging from 18% to 60% with sequences in the databases (Supplemental Fig. 2B). Moreover, about 51.2% of unigenes showed the highest sequence homologies to *Sorghum bicolor*, followed by *Zea mays* (20.4%), which are both members of the *Poaceae* family (Supplemental Fig. 2C).

Gene Ontology (GO) annotation revealed that the unigenes were assigned into 49 functional groups under three major categories i.e., Biological process, Molecular function and Cellular component (Supplemental Fig. 3). The most dominant terms were “cellular component organization and biogenesis” and “metabolic process” in the Biological process category. Within the Cellular component category, “cell”, and “cell part” shared the highest percentage. For the Molecular function category, the most highly represented genes were associated with “binding” and “catalytic activity”. The unigenes were searched against the Eukaryotic Orthologous Groups (KOG) database and were classified into 26 KOG categories (Supplemental Fig. 4). “General function prediction only” and “posttranscriptional regulation, protein turnover, chaperones” were two of the largest clusters, followed by “translation, ribosomal structure and biogenesis” and “signal transduction mechanisms”. “Cell motility” and “unnamed protein” represented two of the smallest categories. Furthermore, the unigenes were mapped to the Kyoto Encyclopedia of Genes and Genomes (KEGG) pathways (Supplemental Fig. 5). In total, 13,524 (17%) unigenes were assigned into 32 KEGG pathways covering five main categories. The largest categories were metabolic pathways associated with “translation”, followed by “signal transduction” and “carbohydrate metabolism”, whereas pathways associated with “signal molecules and interaction” and “sensory system” represented the smallest ones.

### Expression profiles and differentially expressed genes (DEGs)

**Figure 4:**
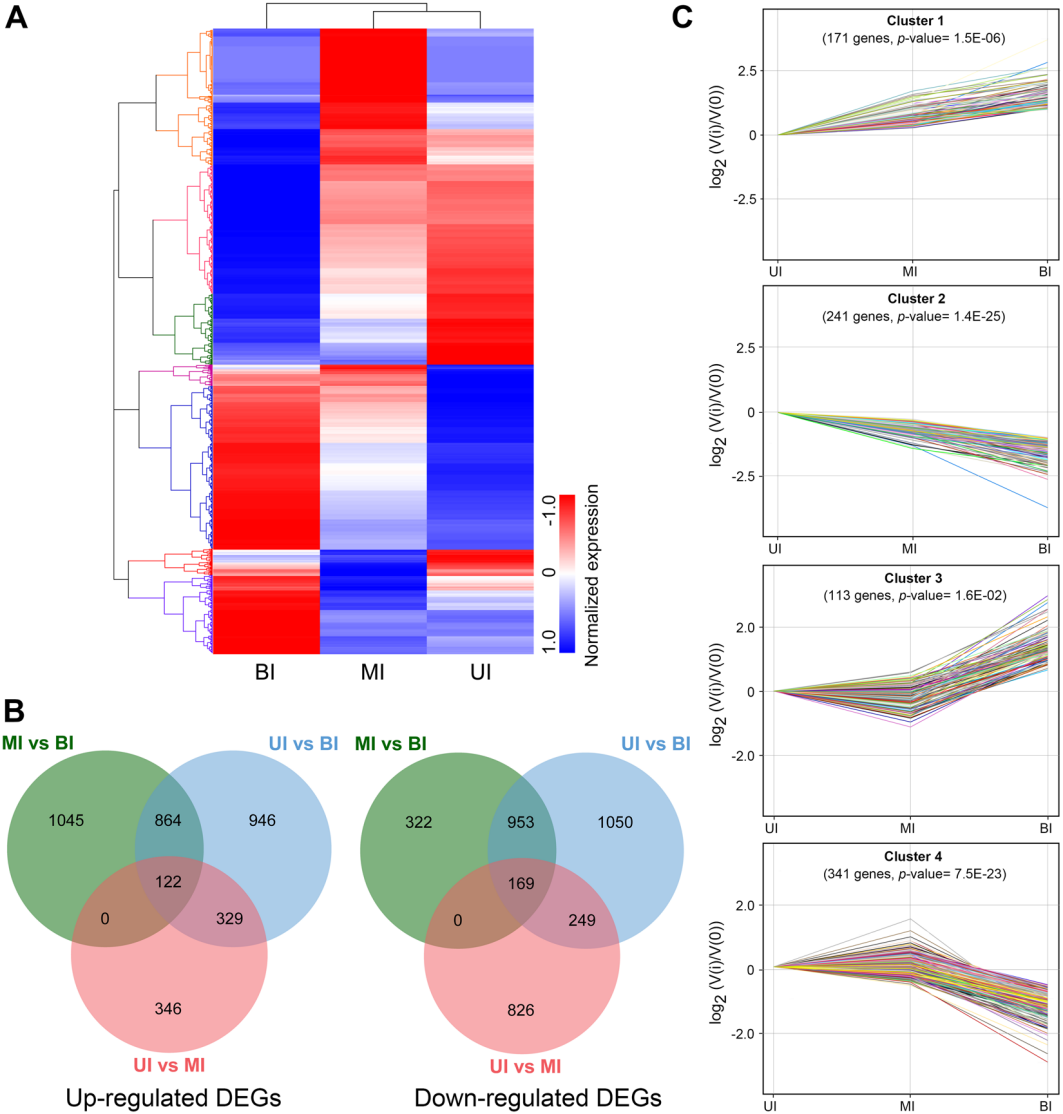
Transcript profiling of genes across three internode segments. (**A**) hierarchical clustering of all differentially expressed genes (DEGs) based on Z-score normalized FPKM values in three internode segments. Blue indicates lower expression, and red indicates higher expression. (**B**) Venn diagram showing the up-regulated or down-regulated DEGs among three internode segments. (**C**) expression patterns of four significantly clustered profiles. UI, upper internode, MI, middle internode, BI, basal internode.

To provide a global view of gene expression across three internode segments, a heatmap was generated using Z-score normalized fragments per kilobase per million (FPKM) values. Hierarchical clustering revealed that the expression could be classified into several distinct patterns (Fig. 4A). To identify genes specifically involved in SCW development, we sought to examine the differentially expressed genes (DEGs) in each internode segment (Fig. 4B). As displayed by the Venn diagrams, 864 genes were significantly up-regulated while 953 genes were down-regulated in the UI and MI compared to the BI, respectively. When compared to the UI, 249 genes were significantly up-regulated, whereas 329 genes were down-regulated in the MI and BI.

K-means clustering analysis was further performed to examine the expression profiles across different internode segments (Fig. 4C). The results showed that the expression profiles were classified into four distinct clusters. The cluster 1 genes showed progressive up-regulation in the MI and BI compared to that of UI. In contrast, genes in cluster 2 displayed contrary expression patterns with progressive down-regulation in the MI and BI compared to the UI. In addition, the expression of genes in cluster 3 and 4 was only significantly up- or down- regulated in the BI compared to that of the UI and MI, respectively. These DEGs may have important functional implications in cell wall development in *M. lutarioriparius*.

### GO and KEGG enrichment analysis of DEGs

**Figure 5:**
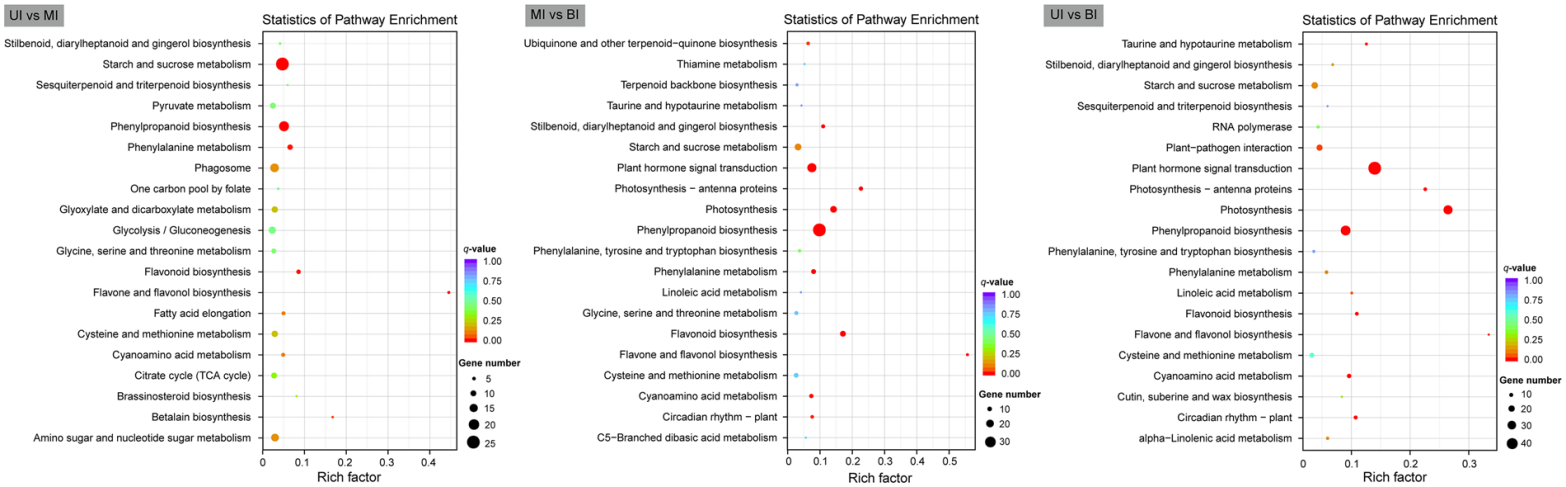
Enrichment analysis of KEGG pathways for differentially expressed genes. Significantly enriched KEGG pathways were plotted for DEGs among the three segments of internode. UI, upper internode, MI, middle internode, BI, basal internode.

To obtain a detailed perspective on the functions of DEGs, we conducted GO and KEGG enrichment analysis. In the Biological process category, genes involved in “carbohydrate metabolic process” and “response to stimulus” had the highest representation in the UI, whereas genes with functions in “organonitrogen compound biosynthetic process” and “single-organism biosynthetic bioprocess” were preferentially present in the MI. Within the Cellular component category, genes associated with “organelle” and “intracellular part” were over-represented in the UI. As for the Molecular function category, genes involved in “(metal) ion binding” and “DNA binding” were highly present in the UI (Supplemental Fig. 6). KEGG pathway enrichment analysis revealed that gene associated with “starch and sucrose metabolism” and “phenylpropanoid biosynthesis” pathways were significantly enriched in the UI versus the MI. Genes involved in “phenylpopanoid biosynthesis”, “plant hormone signal transduction”, and “photosynthesis” pathways were preferentially transcribed in the MI versus the BI (Fig. 5).

**Figure 6:**
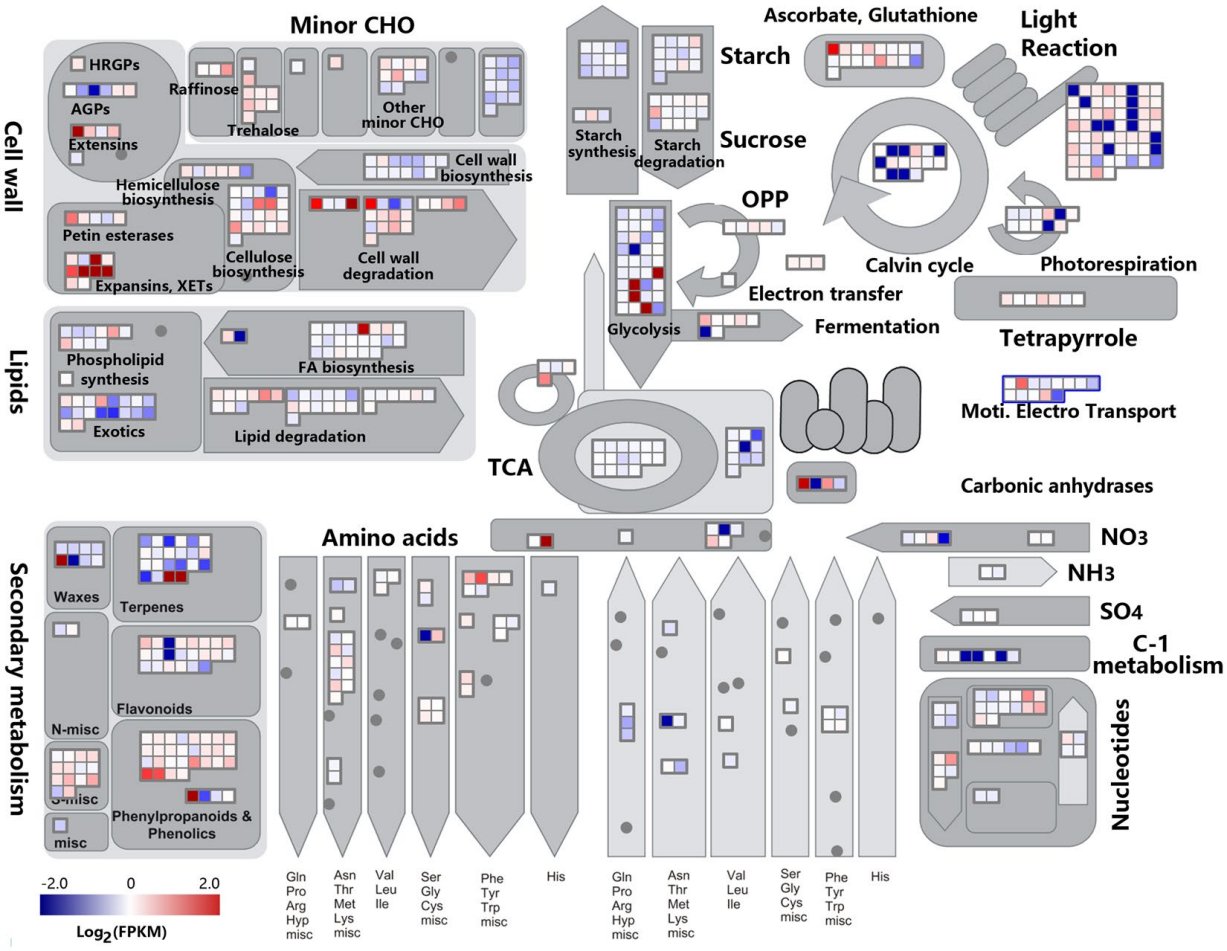
A schematic overview of changes in transcript abundance in the middle internode compared to the upper internode. The log2 transformed FPKM values were imported into MapMan software to generate the overview of general metabolism in the middle internode (MI) compared to the upper internode (UI). DEGs are represented by colored squares and grouped according to functional annotation based on MapMan ontology. The fold change of DEGs is indicated by the scale bar. Red indicates up-regulation, whilst blue indicates down-regulation.

To visually illustrate the transcriptional dynamics during cell wall development in the elongating internode, DEGs were categorized into the metabolic pathways using the MapMan^45, 46^. The results were in good agreement with the aforementioned GO and KEGG analysis. For instance, genes associated with pathways including Calvin cycles, light reactions, and glycolysis etc., were abundantly transcribed in the MI compared to the UI (Fig. 6). The dynamic changes in metabolic pathways for BI versus UI, and BI versus MI were provided as Supplemental Figs 7 and 8, respectively. These results indicated that the transcriptional machinery is under accurate control in different segments of the elongating internode. In such a scenario, cell division and differentiation, and transcriptional regulation is active in the UI, while carbohydrate metabolism and SCW biosynthesis is active in the MI. In addition, SCW deposition and carbohydrate recycling is active in the BI.

Cell wall biogenesis is a very complicated process which incorporates not only multiple gene families directly involved in the polysaccharide biosynthesis and the remodeling of different cell wall components, but also structural proteins involved in cell wall assembly and rearrangement. In addition, cell wall biosynthesis is also under tight transcriptional regulation mediated by specified TFs. We next focused on the detailed expression patterns of gene families involved in SCW biosynthesis and modifications in *M. lutarioriparius*.

### *CESA* and *CSL* gene family

**Figure 7:**
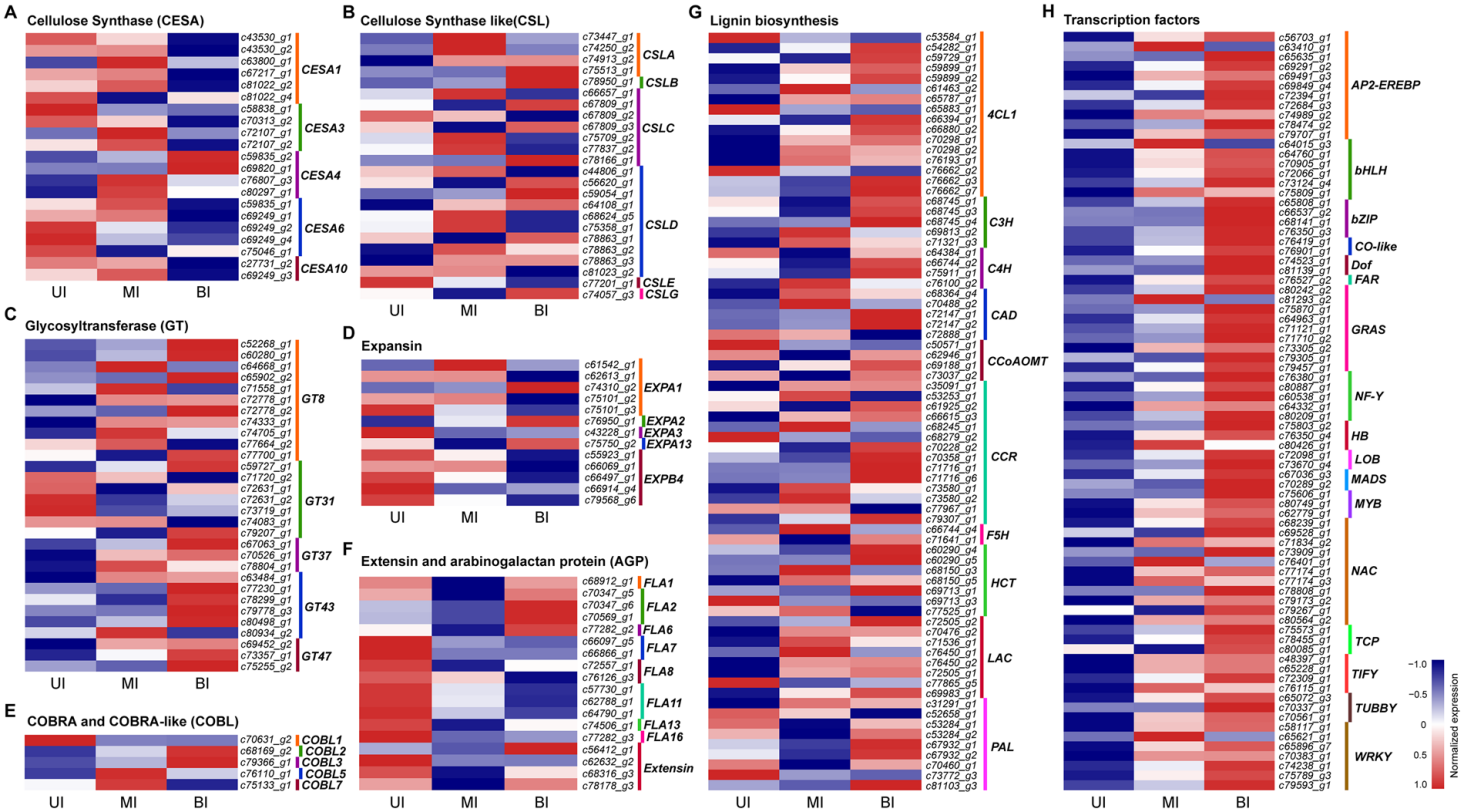
Expression profiles of cell wall biosynthesis genes and transcription factors during internode development. The heatmap was generated with Z-score normalized FPKM values. Up-regulated genes are shown in red, whilst down-regulated genes are in blue. (**A**) expression profiles of cellulose synthase genes. (**B**) expression patterns of cellulose synthase-like genes. (**C–F**) expression analysis of genes encoding cell wall structural proteins. (**G**) expression profiles of monolignol biosynthesis genes. (**H**) expression patterns of transcription factor genes. UI, upper internode, MI, middle internode, BI, basal internode.

*CESA* and *CSL* genes are members of the GT2 family, which are mainly involved in the biosynthesis of cellulose and hemicellulose components in plants^11, 13–15^. Totally, 21 members of *CESA* genes were identified to be differentially expressed in different internode segments (Fig. 7A). Among them, four members homologous to *Arabidopsis CESA4*, which is an essential component of the CESA complex in SCWs, were highly expressed in the MI and BI compared to the UI. In contrast, 15 members homologous to the primary cell wall synthesis-related components *CESA1*, *CESA3* and *CESA6* in *Arabidopsis* were preferentially expressed in the UI compared to the MI and BI. These results indicated that these members may be involved in the biosynthesis of cellulose in the secondary and primary cell walls, respectively.

*CSL* genes were clustered into eight different subgroups (*CSALA*-*G*)^12^. Twenty-four members of *CSL* genes were shown to be expressed in the three internode segments examined (Fig. 7B). Four *CSLA* genes showed higher expression in the MI and BI and lower levels in the UI. In addition, three members of *CSLC* genes and four *CSLD* genes were preferentially expressed in the MI, whereas the expression of four *CSLD* genes and two members of *CSLB* and *CSLG* were significantly higher in the BI. These results suggested putative roles of these *CSL* genes in the biosynthesis of different hemicellulosic components.

### GT gene family

Besides the GT2 family, members from several other GT families are also implicated in the biosynthesis of cell wall polysaccharides^17–23^. In particular, several members from the GT8, GT43, GT47 and GT61families have been demonstrated to be involved in glucuronoarabinoxylan (GAX) biosynthesis and modification in grasses^47, 48^. Accordingly, 30 members from these gene families were expressed in the three sections of the internode (Fig. 7C). A subset of genes including 11 GT8 members, six GT43 members and three GT47 members were preferentially expressed in the MI and BI compared to the UI, thus represent most likely candidates involved in the biosynthesis of GAX in SCWs in *Miscanthus*. In contrast, four genes belonging to GT31 family were exclusively expressed in the UI, therefore represent putative candidates associated with the biosynthesis of primary cell wall components.

### Structural proteins

Expansins are considered one of the most important regulators of cell wall expansion and loosening during plant cell growth^49^. Transcripts of 13 expansins were relatively higher in the UI and MI, which is consistent with their functions as cell wall loosening regulators during cell elongation (Fig. 7D).

*COBRA* family genes encode glycosylphosphatidylinositol (GPI)-anchored proteins that are involved in the assembly of crystalline cellulose during SCW formation^50^. Four *COBRA-like* (*COBL*) genes were predominantly expressed in the MI and BI compared to the UI (Fig. 7E). Their expression patterns highly resembled those of several *CESA* genes (Fig. 7A), indicating that they might be cooperatively involved in cellulose biosynthesis and assembly in SCW development.

Extensins and arabinogalactan-proteins (AGPs), which represent two types of hydroxyproline-rich glycoproteins (HRGPs), play essential roles in the assembly and modification of plant cell wall^27^. Most of the extensin family members were preferentially expressed in the UI compared to the MI and BI, suggesting a putative role in the assembly of primary cell wall (Fig. 7F). The fasciclin-like AGPs (FLAs) are particularly involved in cell wall modification and assembly^27^. A majority of *FLA* genes showed preferential expression in the UI compared to the MI and BI, whereas four genes annotated as *FLA2* or *FLA6* homologs were expressed at higher levels in the BI (Fig. 7F), suggesting their different roles in cell wall modification during internode growth.

### Lignin-related phenylpropanoid genes

Lignin biosynthesis is a complex process that involves many enzymatic reactions^24^. A total of 74 genes involved in lignin biosynthesis pathways were differentially expressed in three internode segments (Fig. 7G). The transcript abundance of these genes varied substantially and exhibited various expression patterns. As expected, the transcript levels of several lignin biosynthesis genes including four *phenylalanine ammonialyase* (*PAL*), nine *4-coumarate-CoA ligase* (*4CL*), two *caffeoyl CoA 3-O-methyltransferase* (*CCoAOMT*), four *cinnamyl alcohol dehydrogenase* (*CAD*), and eight *cinnamoyl-CoA reductase* (*CCR*), and six *laccase* (*LAC*) genes were relatively lower in the UI, but were dramatically increased in the MI and BI. The expression patterns of these genes were consistent with the active SCW biosynthesis and substantial lignification in the MI and BI sections. In contrast, several genes encoding enzymes involved in the early steps of phenylpropanoid metabolism, including three *PAL* genes and three *4CL1* genes were predominantly expressed in the UI. These genes might be responsible for the biosynthesis of phenylpropanoid derivatives rather than lignin components, which serve as important barriers in defense against pathogen infections.

### Transcription factors

To provide insight into the transcriptional regulation of SCW development, the differential expression of TFs in three internode segments was examined. The results showed that a total of 78 TFs belonging to 17 different families were preferentially expressed in the MI and BI compared to the UI, suggesting putative roles in the regulation of SCW formation (Fig. 7H). The largest families include NAC (11 members) and AP2-EREBP (11 members), followed by GRAS (9 members) and WRKY (7 members).

### Co-expression network

**Figure 8:**
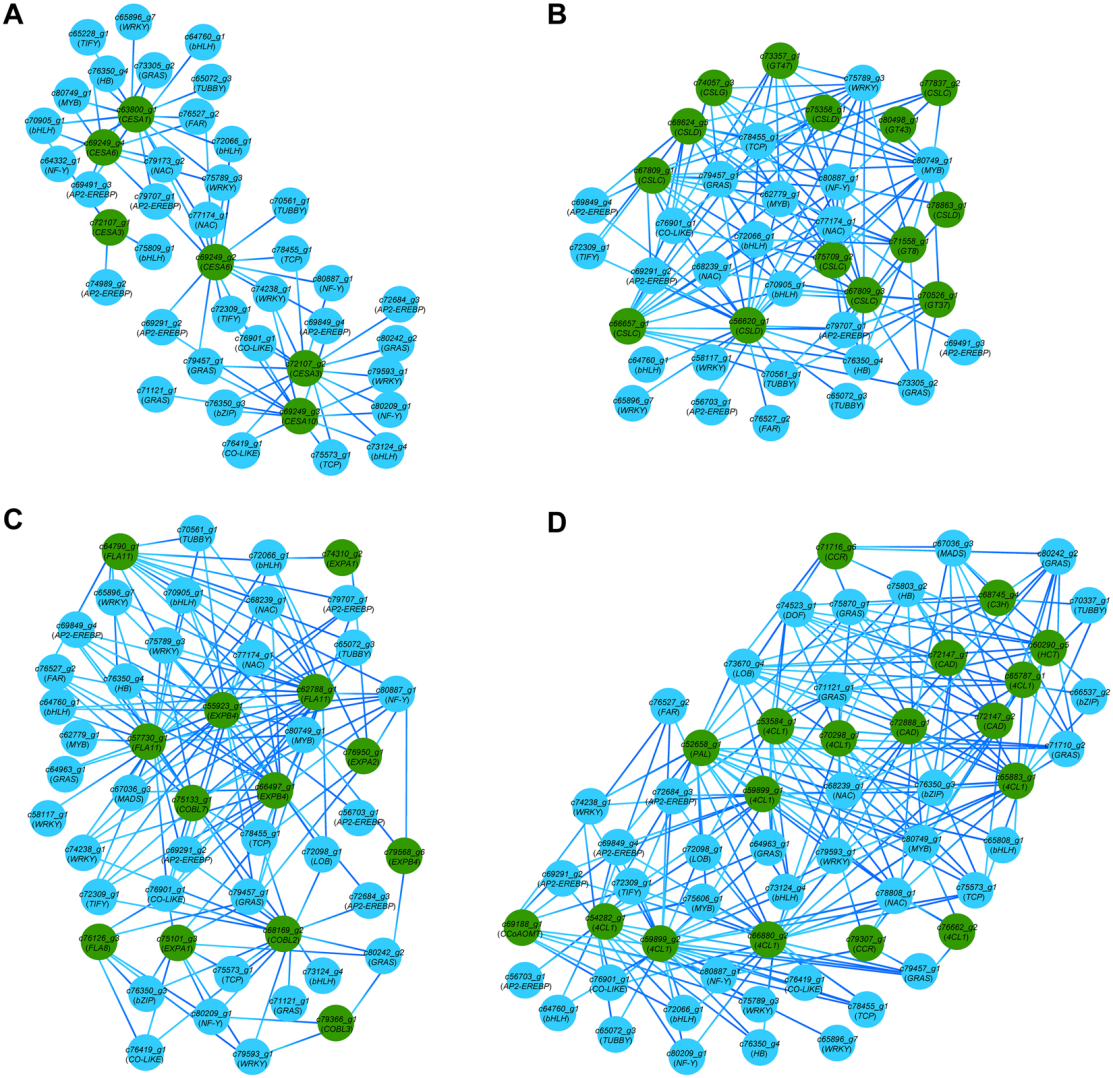
Co-expression network of transcription factors with secondary cell wall biosynthesis genes. Regulatory networks showing transcription factors co-expressed with 6 cellulose synthase genes (**A**), 14 hemicellulose biosynthesis genes (**B**), 13 genes encoding cell wall structural proteins (**C**), and 18monolignol biosynthesis genes (**D**). Transcription factor genes are in blue color and cell wall component biosynthesis genes are in green color.

Co-expression analysis has been proven to be an effective and powerful means for the detection of functional gene modules underlying specific biological processes. In this respect, we constructed the co-expression networks of TFs and SCW biosynthesis-related genes to identify TFs that are specifically involved in the regulation of individual SCW component (Fig. 8). The results showed that 36 TFs were directly connected to the six cellulose biosynthesis genes. Most of these TFs belong to the AP2-EREBP, bHLH, and NAC families. A total of 25 TFs from 12 families were revealed to be associated with 14 unigenes involved in hemicellulose biosynthesis. Most of these TFs are from the AP2-EREBP, WRKY and NAC families. There are 36 TFs associated with 13 unigenes encoding cell wall structural proteins. These TFs belong to 15 different families including WRKY, NAC and MYB etc. In addition, 40 TFs were directly connected with 18 genes involved in monolignol biosynthesis. These TFs are generally members of the GRAS, WRKY, and NAC families. These results indicated that these TFs are most likely important regulators in the biosynthesis of specific SCW components. Remarkably, a substantial number of TFs have multiple connections with genes involved in the biosynthesis of different cell wall components, indicating that these TFs might synergistically regulate the biosynthesis of different SCW components.

### Verification of DEGs expression by qRT-PCR

**Figure 9:**
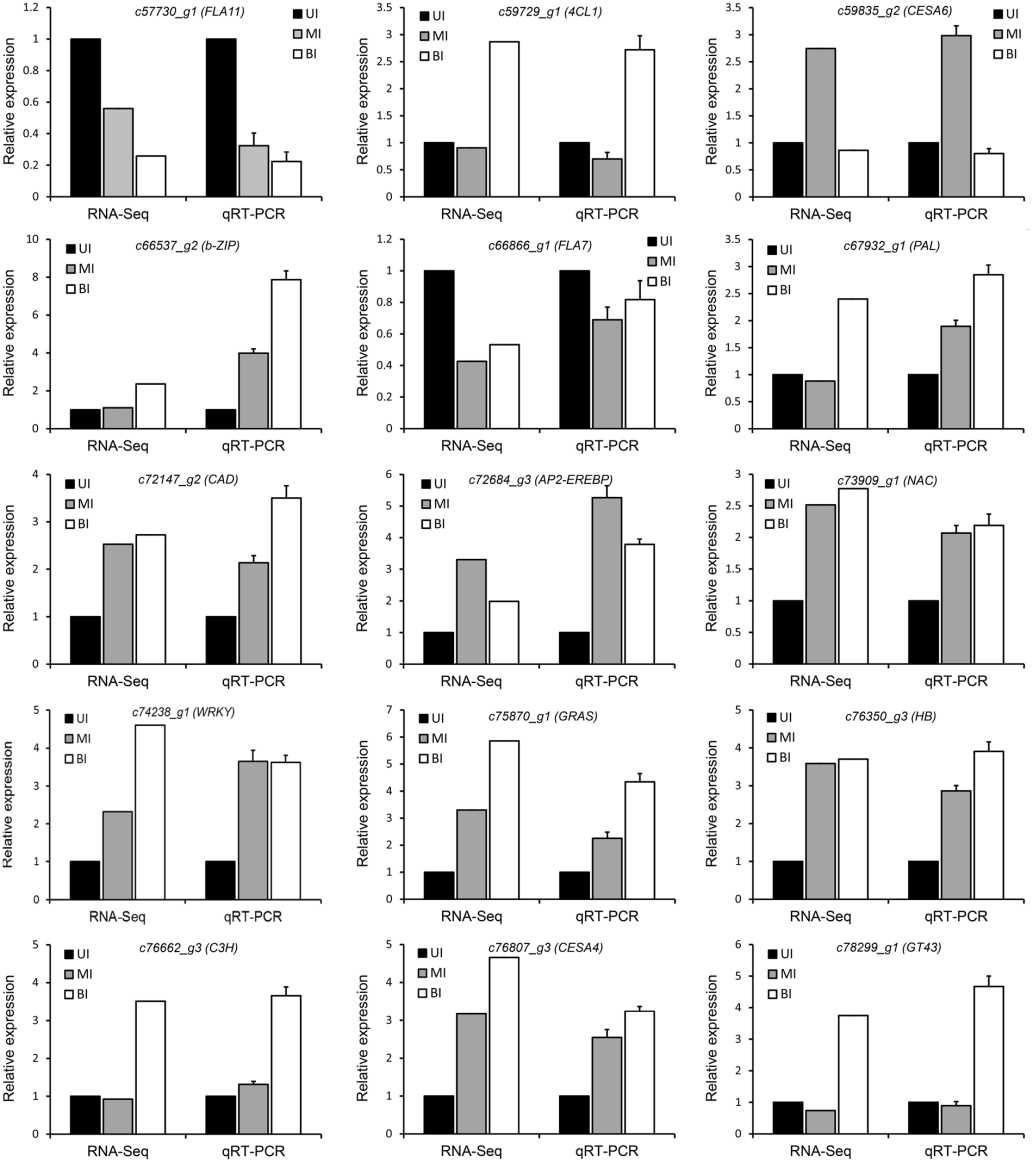
Validation of gene expression by quantitative real time RT-PCR. The expression of the UI was set as 1.0, and the relative expression level was calculated. UI, upper internode, MI, middle internode, BI, basal internode.

To validate the expression of SCW biosynthesis genes obtained by the RNA-seq, quantitative real-time RT-PCR (qRT-PCR) analysis was carried out. The expression of 15 genes and TFs involved in cellulose, hemicellulose and lignin biosynthesis was examined across three internode segments. The results showed that the relative transcript levels of the genes examined by qRT-PCR were in good agreement with the RNA-seq (Fig. 9), which confirmed the robustness and reliability of the RNA-seq results.

## Discussion

*Miscanthus* (particularly *M. lutarioriparius*) is a promising lignocellulosic bioenergy crop. Its superior high biomass yield, low fertilizer requirement and wide adaptability to marginal land make it suitable as a feedstock for the next generation bioethanol production. The compositions of SCW and the cross-linking among the components significantly influence the saccharification efficiency of lignocellulosic biomass into fermentable sugars^51–53^. Thus, a better understanding of the biosynthesis and regulation of SCW is of fundamental importance to the efficient utilization of lignocellulosic biomass. In this study, we carried out a detailed transcriptome analysis of SCW biosynthesis during the growth of an elongating internode using RNA-seq. We described the global transcriptional profiles of genes involved in the biosynthesis of SCW polysaccharides, structural proteins and lignin polymers. Meanwhile, we also examined the expression of TFs and constructed co-expression networks involving TFs and genes responsible for SCW biosynthesis and modification.

Grass cell walls differ dramatically with those of dicot plants with respect to the major polysaccharide compositions, the patterns of cross-linking and the abundance of structural proteins etc^54^. For example, xyloglucans are predominantly present in the primary cell walls of dicots and the non-commelinoid monocots, whereas in grasses, xyloglucans are dramatically reduced and partially replaced by glucuronarabinoxylans^55^. In addition, the abundance of pectins and structural proteins are usually lower in grasses compared to that of dicots and non-commelinoid monocots^54^. *Miscanthus* has typical type II cell walls similar to grasses. As revealed by previous studies, the lignicellulosic biomass of *Miscanthus* is composed of approximately 32.7–49.5% cellulose, 21–34.8% hemicellulose, and 17.8–27.7% lignin^1, 2, 56–59^. The majority type of hemicellulosic components of *Miscanthus* is arabinoxylan^56, 59, 60^. Meanwhile, a minor proportion of mixed linkage β-glucan is also present^60^.

In the dicot model plant *Arabidopsis thaliana*, recent years have witnessed an ever increasing reports on the functions of cell wall biosynthesis genes, most of which were revealed by reverse genetics approaches^33, 61^. In contrast, functional studies of genes involved in cell wall biosynthesis are largely lagged behind in *Miscanthus*. Usually, the functionality of new identified cell wall genes in grasses can only be inferred from the information available from *Arabidopsis* or rice based on sequence homology searches. However, because of the distinctive differences in cell wall compositions and the interaction among cell wall polymers between grass and dicot plants, the functionality usually could not be accurately inferred just simply based on sequence homology^39, 62^. Moreover, our results showed that up to 30% of *M. lutarioriparius* genes exhibited sequence similarities below 40% and E-values lower than 1E-30 when BLAST against the non-redundant proteins available in NCBI (Supplemental Fig. 2). Similarly, a genome-wide comparative analysis of gene families in maize with that of sorghum, rice and *Arabidopsis* revealed that more than 17.5% of genes families are grass-specific and have no functional orthologs in *Arabidopsis* ^63^. With respect to cell wall-related genes, it has been estimated that more than 30% of grass genes have no orthologs in *Arabidopsis*. In addition, accumulating evidence indicated that the closest homologous genes of *Arabidopsis* might perform divergent roles in cell wall development in grasses (e.g., switchgrass) ^64^. Therefore, it is essential to set up a functional genomics platform for grasses. Currently, the whole genome sequence of *Miscanthus* is not yet available, thus makes the genome-wide study of cell wall biosynthesis genes in *Miscanthus* almost impossible. However, with the advancement of next-generation sequencing technique, it is applicable to provide insight into cell wall biosynthesis in *Miscanthus* by *de novo* transcriptome analysis via RNA-seq.

Transcriptome analysis has been proven to be a very powerful means in mining genes involved in specific biological processes especially for species without reference genome sequences. Transcriptome analysis has also been widely employed to identify genes involved in SCW biosynthesis in diverse plant species including *Arabidopsis* ^65^, poplar^66^, maize^38, 39^, switchgrass^42^, sugarcane^67^, sorghum^40^, *Medicago* ^41^, and *Setaria viridis* ^68^. Generally two types of sampling strategies were employed in the dissection of stem segments in the transcriptome analysis. Several studies compared the transcriptome between the elongating and un-elongating internodes, examples including *Arabidopsis* ^65^, poplar^66^, *Medicago* ^41^, maize^39^, sorghum^40^ and sugarcane^67^ etc. In contrast, comparative transcriptome analysis were also carried out by utilizing different stem segments from an elongating internode in switchgrass^42^, maize^38^ and *Setaria viridis* ^68^. Both sampling strategies have their advantages and disadvantages. The advantage of the former sampling method is that the cell wall development state of the sample is dramatically divergent and could possibly fully cover all the DEGs. However, just because of the large differences that might include differences besides cell wall development among stem segments (or internodes), the DEGs identified might contain a considerable number of genes that are not related to cell wall development. Just because of the background noise of the former sampling method, some *bona fide* genes involved in the development of cell wall might also be masked or filtered out. As for the latter sampling method, in which different segments of an elongating internode was utilized, the differences among the segments were mainly confined to the cell wall development state. Therefore the background noise could be significantly reduced, and it can be assumed that the DEGs involved in cell wall development could be efficiently identified. From this perspective, the latter sampling method may have certain advantages over the former one. Moreover, the utilization of different sections of an elongating internode has been proposed as a model system to study plant cell wall development^42, 68^.

The transcriptome analysis showed that almost all the genes involved in cell wall biosynthesis, assembly and modification have orthologs in *M. lutrarioriparius* (Fig. 7). However, distinctive differences in term of gene numbers and expression patterns were observed as compared to *Arabidopsis*. For example, a larger number of genes belonging to GT8, GT43 and GT47 families were present in *M. lutarioriparius* compared to *Arabidopsis*. These GT genes are mainly responsible for the biosynthesis of arabinoxylan, the predominant hemicellulosic components in *M. lutarioriparius* ^69^. The over-representation of these gene family members might due to the fact that arabinoxylan is present in both primary and secondary cell walls of *M. lutarioriparius* ^56, 59^. In contrast, glucoarabinoxylan, as the main hemicellulosic component, is only abundantly present in SCWs in *Arabidopsis* ^54^. In addition, a subset of genes exhibited distinctive expression patterns as compared to their closest orthologs in *Arabidopsis* (Fig. 7). These genes might be involved in grass-specific cell wall-related processes.

TFs play pivotal roles in the regulation of SCW biosynthesis, and considerable progress has been made for a number of NAC and MYB TFs. NAC secondary wall thickening promoting factors (NSTs) act as the main switch in SCW regulation, which orchestrate a large number of downstream MYB TFs and SCW biosynthesis genes^34, 70^. Besides NAC and MYB TFs, class III Homeodomain-Leucine Zipper (HD-ZIP III) and WRKY family TFs have also been implicated to play important roles in SCW formation^71, 72^. As expected, our transcriptome profiling analysis revealed that the expression of a large number of TFs belonging to NAC, MYB, homeodomain box (HB) and WRKY families were significantly up-regulated in the MI and BI, suggesting their putative roles in regulating SCW biosynthesis (Fig. 7H). In addition, we also identified several TFs belonging to AUX/IAA, AP2-EREBP and C2H2 zinc finger families that were highly expressed in the MI and BI (Fig. 7H). Similarly, it has been shown that the expression of AP2-EREBP and C2H2 zinc finger TFs was significantly increased during fiber cell wall thickening in cotton^73^, and during SCW biosynthesis in the elongating internode in maize^39^. Furthermore, AP2-EREBP TFs have also been proposed to play a regulatory role in SCW formation in *Arabidopsis* and poplar^74, 75^. In addition, AUX/IAA TFs have been implicated to play important roles in regulating secondary growth in *Arabidopsis* ^76^. Therefore, these families of TFs might also be involved in regulating SCW biosynthesis in *M. lutarioriparius*. But their detailed roles in SCW development awaits further functional characterization by genetic approaches.

Furthermore, TFs have also been revealed to play important roles in regulating plant responses to diverse abiotic and biotic stresses. Meanwhile, plant cell wall serves as a barrier in the defense against various pathogens and abiotic stresses, conferring plant intrinsic resistance to stress conditions. However, it remains to be elucidated if TFs have interacting roles during the transcriptional regulation program in stress resistance and cell wall development.

## Conclusions

In this study, we performed *de novo* transcriptome analysis of an enlongating internode to identify genes involved in SCW development in *M. lutarioriparius*, an important bioenergy crop with high lignocellulosic biomass yield. The histological and *in-situ* histochemical analysis revealed that the elongating internode can be divided into three segments with distinct SCW development states: the UI with abundant primary cell walls, the MI undergoing substantial cell wall thickening, and the BI with predominant SCWs deposited. Transcriptome profiling analysis among the UI, MI and BI led to the identification of genes involved in the biosynthesis of SCW components including cellulose, hemicellulose (arabinoxylan) and lignin, as well as minor structural proteins and TFs involved in cell wall assembly and modification. Furthermore, putative regulatory networks involving TFs and SCW-associated genes were constructed based on gene co-expression patterns. The work presented is the first transcriptome analysis of SCW development in *M. lutarioriparius*. These findings shed new light on the biosynthesis and regulation of SCW in grasses. The genes identified provide excellent candidates for further functional characterization and genetic improvement of lignocellulosic biomass properties toward increased saccharification efficiency.

## Methods

### Plant materials

*M. lutarioriparius* plants were grown in a growth chamber at 25 °C with 16 h light / 8 h darkness photoperiod and 60–70% relative humidity. The second internode counted from the top at stage E11 (elongating-stem comprising 11 internodes) was harvested from six plants representing two biological replicates. The average length of the internodes collected is 91.2 mm, which is approximately 40% of the maximum length at anthesis. After removal of leaves and sheaths, the internode was equally dissected into the upper, middle and basal segments, excluding 1 cm adjacent to each side of the internode. The dissected segments were immediately frozen in liquid nitrogen for RNA extraction, or fixed for histological analysis.

### Histological analysis

Samples were fixed in FAA (3.7% formaldehyde, 5% glacial acetic acid and 50% ethanol) under vacuum for 24 h, dehydrated in a gradient series of ethanol (70%, 85%, 95% and 100%), and embedded in paraffin (Sigma-Aldrich). Sections of 8-μm thickness were cut and stained with 0.05% Toluidine Blue O. Observations were made using an OLYMPUS BX51 light microscope within 5 min of staining.

For phloroglucinol-HCl staining of lignins, sections were treated with 3% (w/v) phloroglucinol solution for 1 min, and then washed with 50% HCl. The slides were mounted in glycerol and observed immediately with an OLYMPUS BX51 light microscope.

For immunolabeling of LM10, sections were firstly incubated with PBS containing 3% (w/v) fat-free milk powder (MP/PBS) for 30 min, followed by incubation in 10-fold dilution of LM10 in MP/PBS for 1.5 h. After brief washes with PBS, sections were incubated with a 200-fold diluted AlexaFluor488-tagged goat anti-rat IgG in MP/PBS in darkness for 1 h. Then sections were washed with PBS and counterstained with Calcofluor White (Sigma-Aldrich) for 5 min. Photographs were taken using a FluoView FV1000 confocal laser scanning microscope (OLYMPUS) under 405 nm and 488 nm lasers.

For immunolabeling of CBM3a, sections were firstly blocked in MP/PBS for 30 min, then incubated in MP/PBS with CBM3a (10 mg ml^−1^) for 1.5 h, followed by incubation in a 100-fold diluted mouse anti-His monoclonal antibody in MP/PBS for 1 h. After washes with PBS, sections were incubated in a 200-fold diluted AlexaFluor488-tagged goat anti-mouse IgG in MP/PBS for 1 h in darkness. The counterstaining with Calcofluor white and microscope observation is the same as that of LM10 previously described.

### RNA extraction

Total RNA was isolated using Trizol reagent (Invitrogen) according to the manufacturer’s protocol, and treated with RNase-free DNase I (TaKaRa) to eliminate DNA contamination. The concentration and purity of RNA was measured using Nanodrop 1000 spectrophotometer (ThermoFisher Scientific). RNA integrity was determined using Bioanalyzer 2100 (Agilent). Only RNA samples with 260/280 ratio higher than 1.8, 260/230 ratio higher than 2.0, and RIN (RNA integrity number) values higher than 8.0 were used for the RNA-seq analyses.

### Library construction and RNA-seq

Six libraries with two independent biological replicates were prepared using the Next Ultra Directional RNA Library Prep Kit (NEB) according to the manufacturer’s instructions. Briefly, mRNA was purified from total RNA using oligo dT attached to magnetic beads. Following purification, the mRNA was fragmented and first strand cDNA was synthesized using M-MuLV Reverse Transcriptase (NEB). Subsequently, the cDNA fragments were blunt ended and linked with adaptors. Sequencing was performed using an Illumina HiSeq2500 platform at Novogene Bioinformatics Technology Co., Ltd (Beijing, China).

### Transcriptome assembly

Raw reads were firstly filtered by removing the adapter sequences, low-quality reads (reads with ambiguous bases > 5% and more than 50% bases with Q ≤ 20) to obtain clean reads using custom Perl scripts. The clean reads were mixed and *de novo* assembled using the Trinity program with K-mer size of 25 and the other parameters set as defaulted^77^. In this assembly, clean reads were combined to form longer fragments called contigs. The paired-end reads were then mapped back to the corresponding contigs to detect contigs from the same transcript and calculate the distances among contigs. The consensus sequences without gaps and could not be extended on either end were termed as transcripts. Finally, the transcripts were then assembled into unigenes by removing redundant sequences using TGI Clustering Tool (TGICL)^78^.

### Functional annotation

The unigenes were searched by BLAST against the NCBI non-redundant protein (Nr), NCBI non-redundant nucleotide sequence (Nt), Swiss-Prot, Protein Family (Pfam), Gene Ontology (GO), Eukaryotic Orthologous Groups (KOG), and Kyoto Encyclopedia of Genes and Genomes (KEGG) databases with an E-value cutoff at 1.0E-5.

### Identification of DEGs and clustering analysis

Differentially expressed genes (DEGs) among different tissues were identified with edgeR package using the read counts. Unigenes exhibiting difference of at least two-fold change with *p*-value lower than 0.05 were considered as differentially expressed. The FPKM value was used to assess the expression levels of unigenes. The Venn diagram of DEGs was generated using an online software (http://bioinfo.genotoul.fr/jvenn/). The heatmap was generated with Z-score normalized FPKM values using the online tools available at OmicShare (http://www.omicshare.com/). The clustering analysis was performed based on average pair-wise Pearson correlation with the Short Time-series Expression Miner (STEM) program^79^.

### GO and KEGG enrichment analysis

The GO categories and KEGG pathway enrichment analysis of DEGs was performed using the hypergeometric distribution test compared to the whole-transcriptome background. The GO terms and KEGG pathways with Bonferroni-corrected *p*-values ≤ 0.05 were considered significantly enriched.

### MapMan analysis of DEGs

The *M. lutarioriparius* annotation mapping file for MapMan was generated using the online Mercator program with default parameters^80^. The log2 transformed FPKM values of DEGs were imported into MapMan to visualize the changes in different functional categories^46^.

### Gene co-expression analysis

Weighted Gene Co-expression Network Analysis (WGCNA) was performed to identify clusters of genes with highly correlated expression patterns^81^. The correlation relationships between specified genes were visualized using the Cytoscape software^82^.

### Validation by quantitative real-time RT-PCR

RNA extraction was performed as described above. cDNA was synthesized with 2 μg of total RNA using Superscript III First strand synthesis kit (Invitrogen) with oligo dT primers following the manufacturer’s instructions. Quantitative real-time RT-PCR (qRT-PCR) analysis was performed using a LightCycler^®^ 480 system (Roche) with SYBR Premix Ex Taq II (TaKaRa). *ACTIN11* was used as a reference gene for expression normalization. The gene-specific primers are listed in Supplemental Table 1. All qRT-PCR reactions were carried out with three technical replications. The relative gene expression was calculated using the 2^−ΔΔCt^ method^83^.

### Data availability

The raw RNA sequencing data have been deposited in NCBI Sequence Read Archive (SRA) under accession SRP092425.

## Electronic supplementary material

Supplementary file
