# Supplementary material for: Transcriptome analysis of genes involved in secondary cell wall biosynthesis in developing internodes of Miscanthus lutarioriparius
Source: Sci Rep. 2017 Aug 22;7:9034. doi: 10.1038/s41598-017-08690-8 (PMC5567372; doi:10.1038/s41598-017-08690-8)
Supplement: Supplementary file 1 — Supplementary file [file 41598_2017_8690_MOESM1_ESM.pdf]

Transcriptome analysis of genes involved in secondary cell wall biosynthesis in developing internodes of *Miscanthus lutarioriparius*

Ruibo Hu, Yan Xu, Changjiang Yu, Kang He, Qi Tang, Chunlin Jia, Guo He, Xiaoyu Wang, Yingzhen Kong, Gongke Zhou

Supplementary material

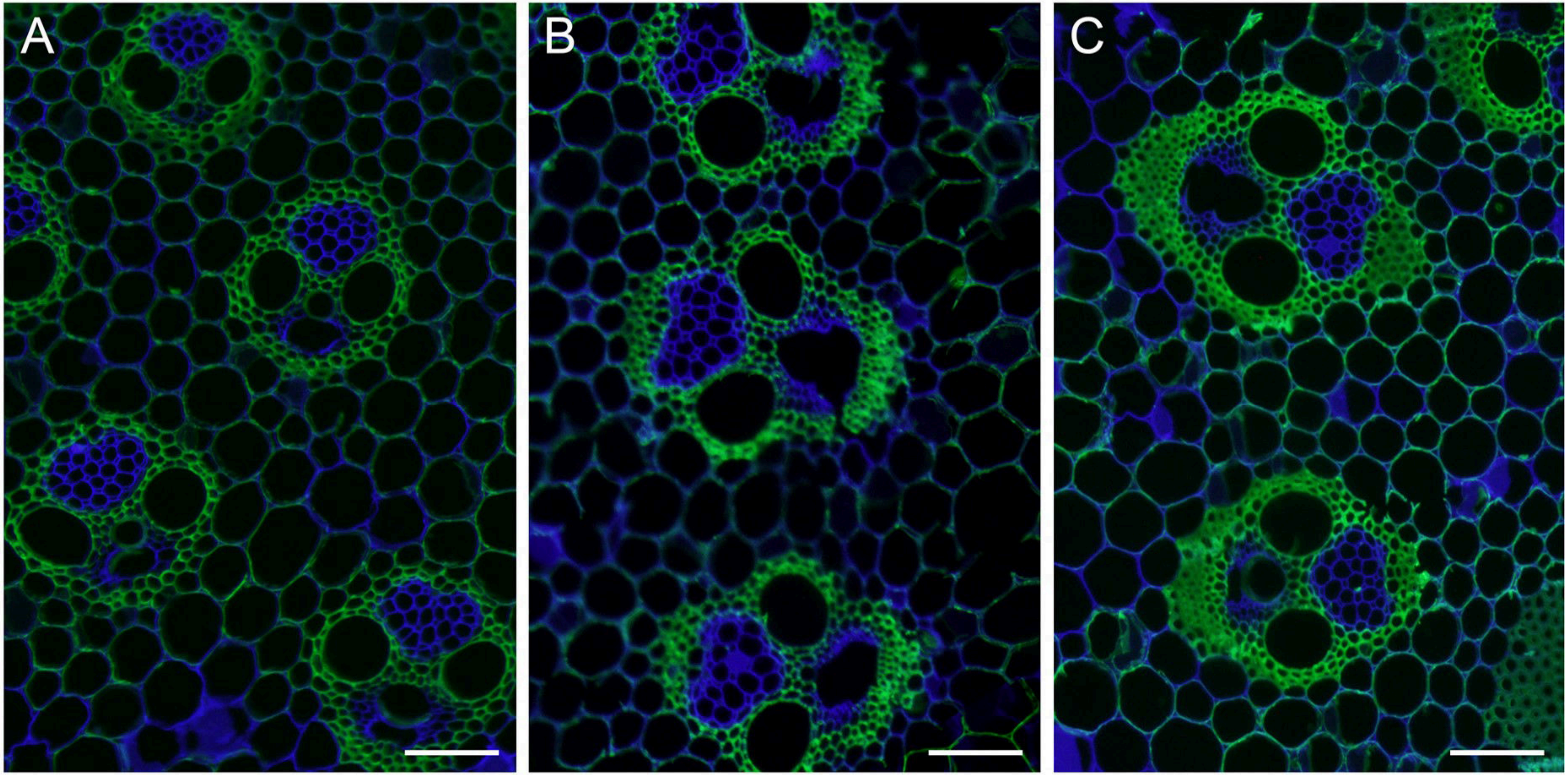

Supplemental Fig. 1. Immuno-detection of (arabino)xylan epitopes in *M. lutarioriparius* internode.

The sections were immunolabeled with LM10 monoclonal antibody and counterstained with Calcofluor. A, upper internode (UI), B, middle internode (MI), C, basal internode (BI). Bar=100 μm.

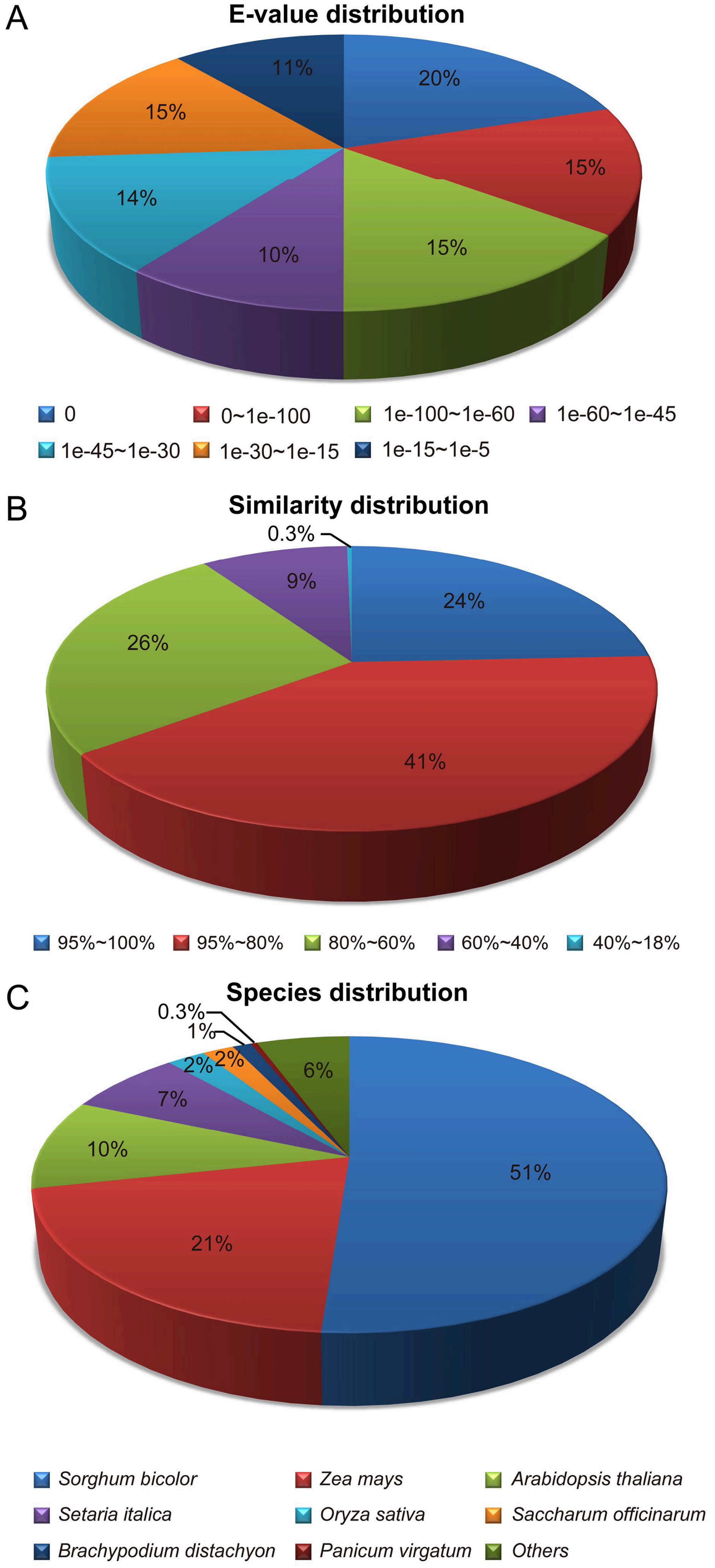

Supplemental Fig. 2. Functional annotation of the transcriptome data.

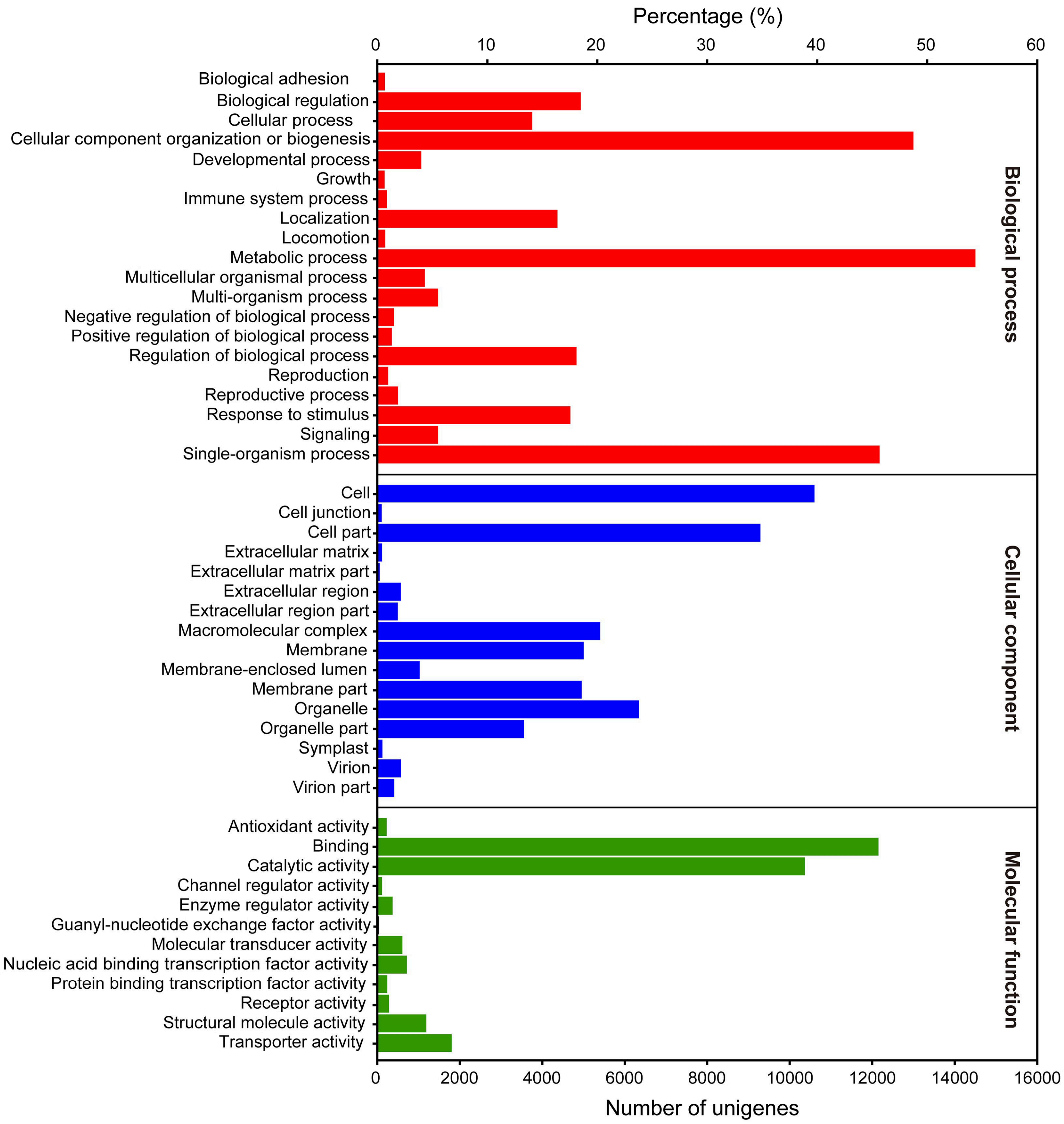

Supplemental Fig. 3. Histogram presentation of GO classification.

Bars represent the numbers and percentages of unigenes matched to GO terms of three categories.

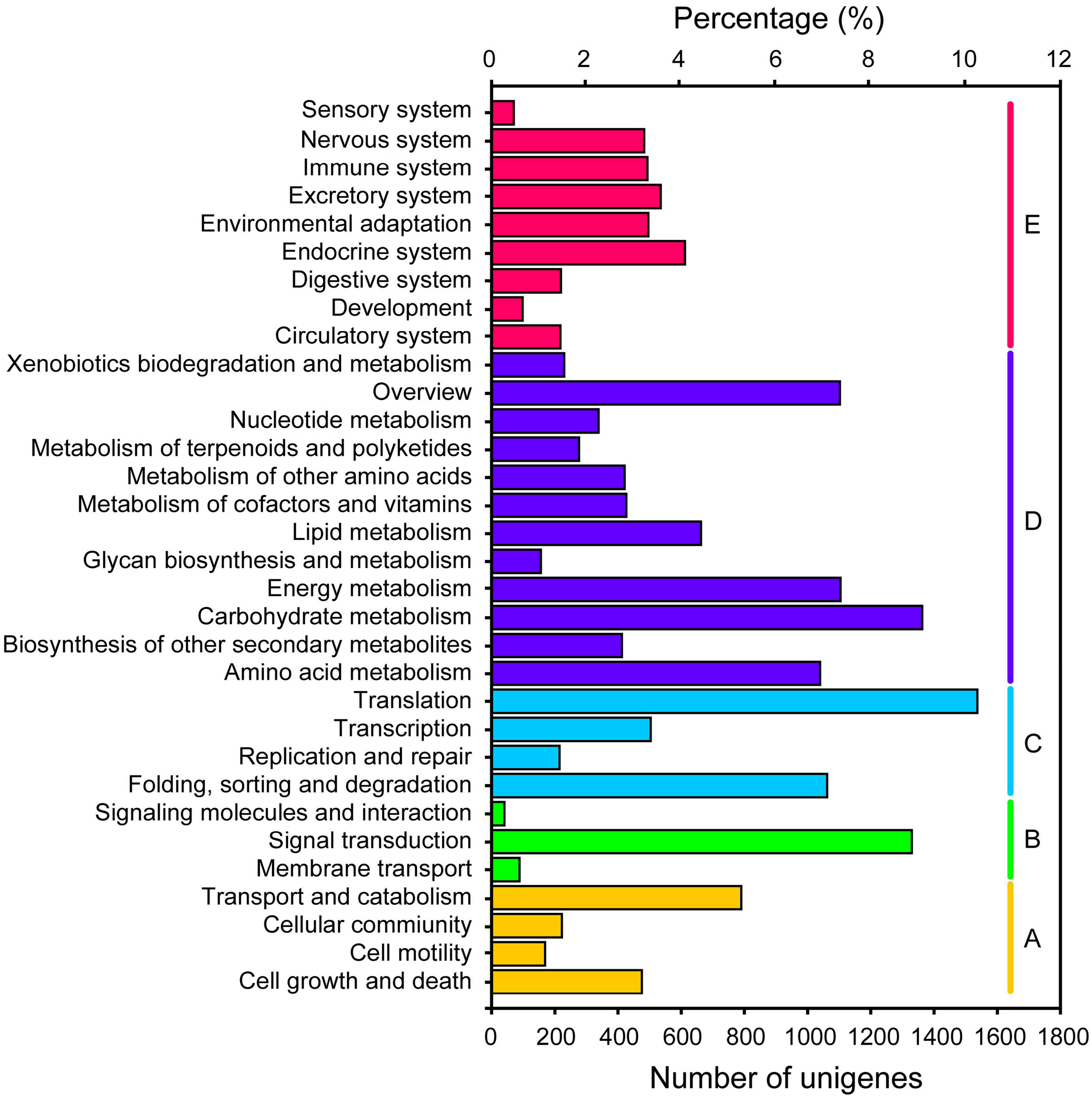

Supplemental Fig. 4. Classification of unigenes in KEGG pathways.

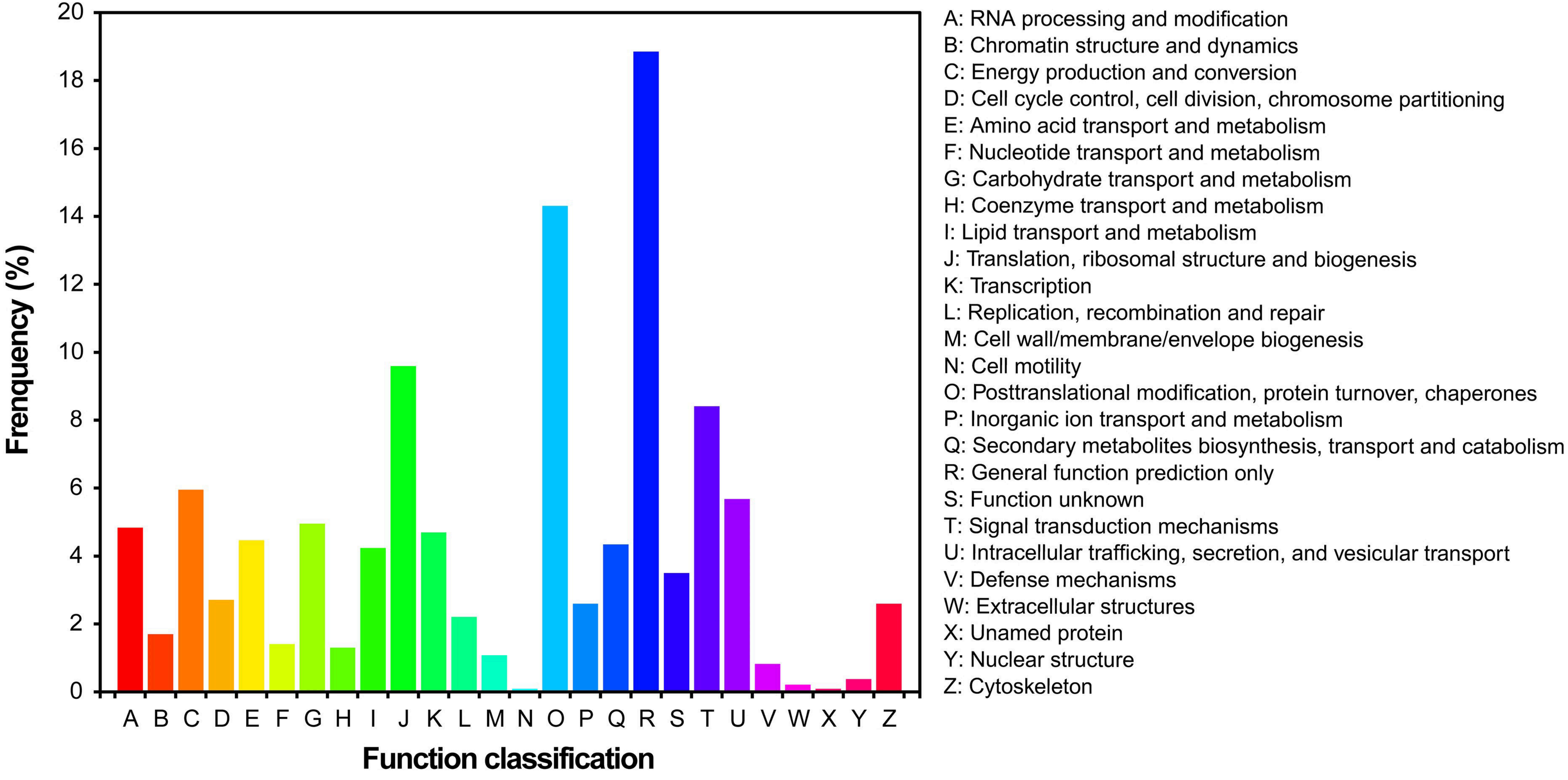

Supplemental Fig. 5. KOG classification of unigenes

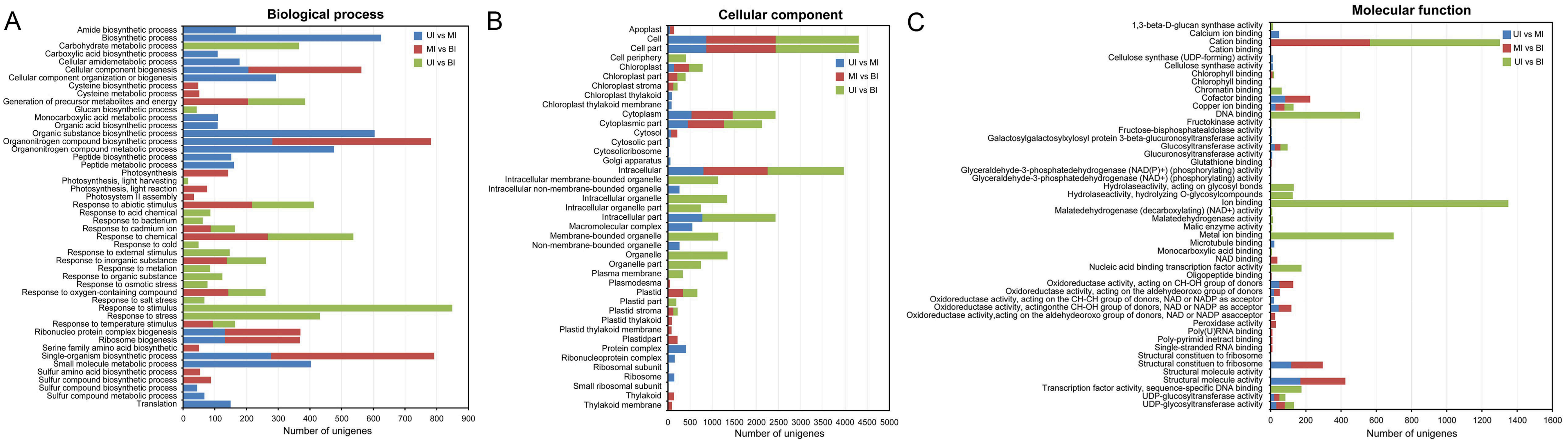

Supplemental Fig. 6. GO enrichment of differential expressed genes among three internode segments.

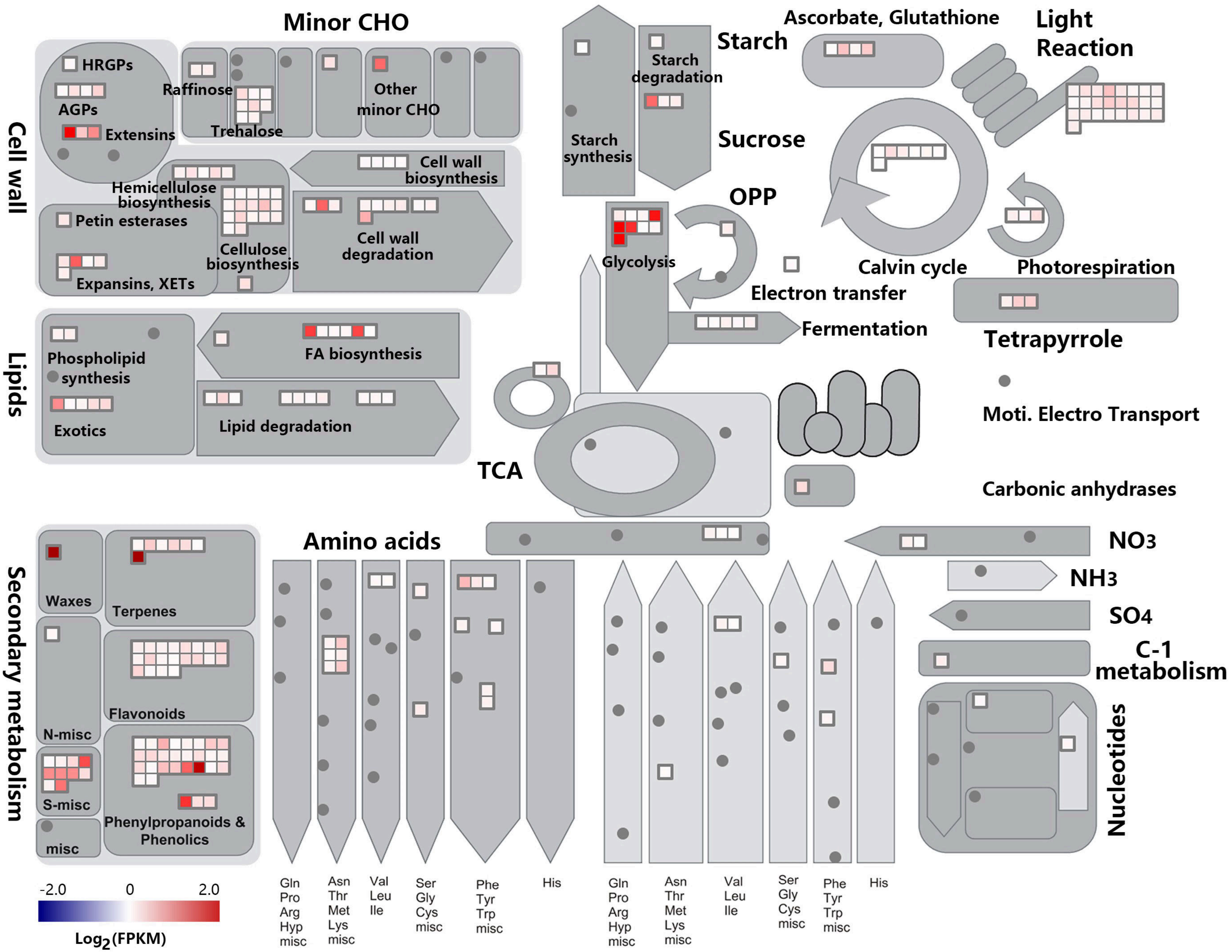

Supplemental Fig. 7. A schematic overview of transcriptional changes in the basal internode compared to the upper internode.

DEGs are represented by colored squares. The fold change is indicated by the scale bar. Red indicates up-regulation, whilst blue indicates down-regulation.

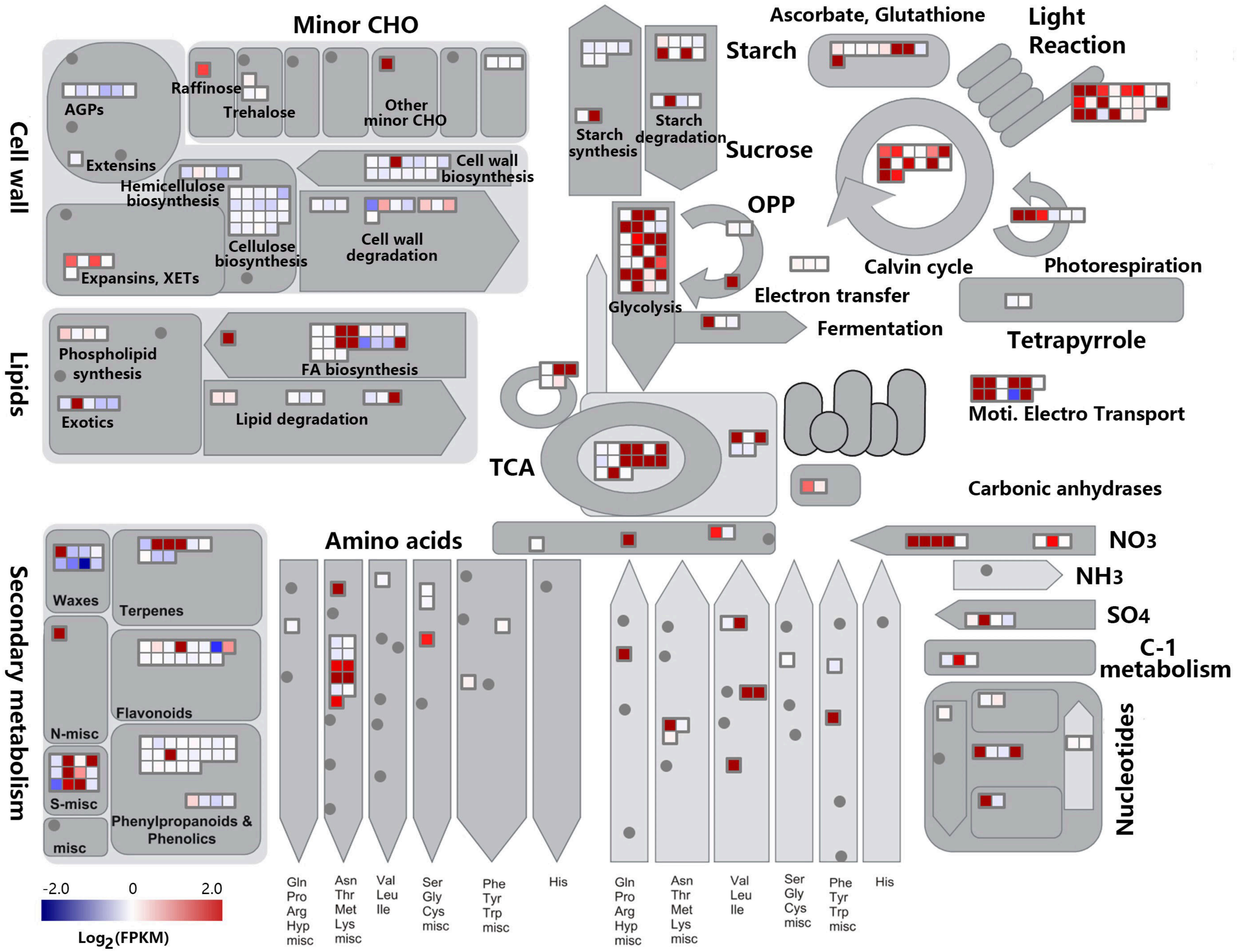

Supplemental Fig. 8. A schematic overview of transcriptional changes in the basal internode compared to the middle internode.

DEGs are represented by colored squares. The fold change is indicated by the scale bar. Up-regulated genes are shown in red, whilst down-regulated genes are in blue.

Table S1. Primer sequences for qRT-PCR analysis.

**Gene_ID**	**Forward primer (5'-3')**	**Reverse primer (5'-3')**
c57730_g1	ATTATTCTTCCCTGCCATT	TGAGACAACGACATTACAT
c59729_g1	ATCTCGTTCTCACTAATCTC	TGTTGTCTCGTAAGTTGAT
c59835_g2	ATGCTGTGCTATATGGTTAT	CCTTGCTGGAGTTATTCA
c66537_g2	ACATTCACCACAAGTTCA	TACTGCTGCTGTCTACTA
c66866_g1	CATTAGCCACTGACTGAA	TACTTGTTGCCTATCTCTG
c67932_g1	CACAACAACATTCACATCA	CAAGAGCAGAGAAGACAA
c72147_g2	GCTTCATCTAATTCACCATAG	TAGTGTTATGTGAGGAAGAATA
c72684_g3	TACGGTTGGAAAGTTCTC	AACACTCTTCTTGATGACA
c73909_g1	ATGTCGTTGTGATGTTGA	GCTTGGAAGAACTGATAATG
c74238_g1	TGAGTGACAACAACCTAC	CTCTGCCTGAAACAAGAT
c75870_g1	CACCATCTCATCCATCTC	GAGCATAACCATCCTTGT
c76350_g3	CATAACAGACAGCACAATAC	CCTTATCCAGCACAGTAAT
c76662_g3	CGAGAGAGTTGACTGTAG	CCTTGATTGATGGAGCAT
c76807_g3	GAGTTTCAGTTTCAGTTTCA	TCCATCAGTCTTCAGAGA
c78299_g1	GTAGTTGTGGTGGTAGTG	CTTCTTCCTGCTCTTCTC
ACTIN11	CTCGTCTTCCTCACCGTTATCAC	GCGTCATCTCCAGCGAACC
